# The Crossroads of Neuroinflammation and Biomarkers in Multiple Sclerosis: A Systematic Review

**DOI:** 10.3390/cells15070610

**Published:** 2026-03-30

**Authors:** Maria-Georgiana Gavrilă, Carmen Valeria Albu, Bogdan Cristian Albu, Emilia Burada, Raluca Elena Sandu, Roxana Surugiu

**Affiliations:** 1 Department of Neurology, University of Medicine and Pharmacy of Craiova, St. Petru Rares, No. 2-4, 200433 Craiova, Romania; mariagavrila95@yahoo.com (M.-G.G.);; 2 Doctoral School, University of Medicine and Pharmacy of Craiova, 200349 Craiova, Romania; 3 Emergency Clinical County Hospital Craiova, 200642 Craiova, Romania; 4 Department of Physiology, University of Medicine and Pharmacy of Craiova, St. Petru Rares, No. 2-4, 200433 Craiova, Romania; 5 Department of Biochemistry, University of Medicine and Pharmacy of Craiova, St. Petru Rares, No. 2-4, 200433 Craiova, Romania

**Keywords:** multiple sclerosis, neurofilament light chain, glial fibrillary acidic protein, biomarkers, neuroinflammation, progression independent of relapse activity, disease-modifying therapies

## Abstract

The management of multiple sclerosis (MS) is shifting from a phenotype-based framework toward a biologically driven precision medicine model, as conventional magnetic resonance imaging (MRI) inadequately captures smoldering inflammation and progression independent of relapse activity (PIRA). This systematic review aimed to synthesize current evidence on the diagnostic and prognostic utility of fluid biomarkers in distinguishing acute inflammatory injury from chronic neurodegeneration. A comprehensive search of Web of Science, PubMed, and Scopus (January 2020–September 2025) identified 28 eligible studies including 7775 participants (6365 MS patients and 1410 controls). Biomarkers derived from serum, plasma, cerebrospinal fluid (CSF), and stool were evaluated in relation to clinical disability measured using the Expanded Disability Status Scale (EDSS) and magnetic resonance imaging (MRI) outcomes. Neurofilament light chain (NfL) consistently predicted acute inflammatory activity, gadolinium-enhancing lesions, and relapse-associated worsening, but levels were reduced by high-efficacy therapies and did not reliably predict PIRA. In contrast, glial fibrillary acidic protein (GFAP) was associated with astrogliosis, disability progression, and retinal thinning, even in patients with low inflammatory activity. Additional CSF, metabolic, and immunologic markers correlated with neurodegeneration and disease severity. Nevertheless, broader clinical use will require greater assay standardization, improved consistency across cohorts, and validation in prospective longitudinal studies. These findings compel a shift toward a multi-biomarker model to guide personalized therapeutic strategies and develop targeted neuroprotective treatments for progressive multiple sclerosis.

## 1. Introduction

Multiple sclerosis (MS) is a chronic, immune-mediated disorder of the central nervous system (CNS), characterized by inflammation, formation of multifocal areas of demyelination, and progressive neurodegeneration, affecting approximately 2.8 million people worldwide [1,2,3].

The most common phenotype of MS is relapsing-remitting multiple sclerosis (RRMS), which represents approximately 85% of cases and has a specific dynamic, being characterized by discrete neurological episodes (relapses), followed by episodes of stability or improvement [4,5].

MS is a highly burdensome disease because disability can accumulate over time and can disrupt daily living, independence, mobility, social participation, and work roles [6]. The disease is overwhelming not only by producing disability, but also by having a high risk of mortality and comorbidities [7]. Because MS usually begins during a highly productive stage of life, it has a strong impact on patients, their families, and society, and although disease-modifying therapies can reduce disability and extend survival, a cure is still lacking [8]. Even when relapses and magnetic resonance imaging (MRI) lesion activity are tracked, they may not fully capture neurodegenerative progression, and disability worsening measured by Expanded Disability Status Scale (EDSS) incompletely reflects key outcomes such as cognitive decline and fatigue [9].

The diagnostic framework for MS has been updated with the 2024 revisions of the McDonald criteria, reflecting continued efforts to improve diagnostic performance and integrate advances in paraclinical testing [10]. Because standard clinical examination or MRI measures can “miss” silent progression, fluid biomarkers are increasingly used to improve clinical assessment and imaging for risk stratification, monitoring, and treatment response [11]. Neurofilament light chain (NfL) provides clinically useful information for prognosis and therapeutic efficacy when it is used alongside MRI and clinical examination [11,12,13]. It is a marker with prognostic value both for short-term and long-term outcomes; higher serum neurofilament light chain (sNfL) levels are correlated with subsequent gadolinium-enhancing lesions or new T2 lesions, relapse risk, brain atrophy, and extended progression timeline [11,14]. At the same time, treated patients who are showing lower levels of NfL after 6 months of treatment have a better prognosis with lower brain atrophy, fewer T2 lesions after 2 years, and less EDSS change at year 4 [11,15]. On the other hand, glial fibrillary acidic protein (GFAP) showed moderate correlations with disability, which makes this marker a candidate marker of progression rather than acute inflammatory activity [16]. Chitinase-3-like protein 1 (CHI3L1) is emerging as a marker of early risk stratification, also being closely associated with progression mechanisms, more so than with relapse-only activity. Higher levels of these markers were identified in patients with progressive forms of MS than in RRMS [17]. Because MS pathology includes inflammatory and neurodegenerative components, an overall pattern of increased oxidative stress markers and reduced antioxidants had been identified [18]. Components of the complement system are currently being investigated as potential biomarkers of inflammation and tissue injury in multiple sclerosis (MS). Emerging evidence suggests that complement activation may also contribute to disease mechanisms and could help differentiate between MS phenotypes [19]. In clinical practice, an integrated multi-biomarker approach could complement MRI and clinical examination by mapping axonal injury, astroglial activation, intrathecal B-cell immunity, complement activity, and redox injury onto patient monitoring and treatment decisions, although assay standardization and longitudinal validation are still needed [11].

This systematic review aims to synthesize current evidence on blood and CSF biomarkers in MS, focusing on their diagnostic and prognostic value, their associations with clinical disability (EDSS), MRI activity and neurodegeneration, and their behavior under treatment, and the translational implications of integrating complementary biomarkers into clinical monitoring and mechanism-based therapeutic strategies.

## 2. Materials and Methods

This review was conducted according to the reporting guidance provided by the Preferred Reporting Items for Systematic Reviews and Meta-Analyses (PRISMA) 2020 statement [20]. The study protocol was registered in the Open Science Framework (OSF) and is publicly available online (https://osf.io/92qdk) (accessed on 3 March 2026). The completed PRISMA 2020 checklist is provided in Supplementary Material S1.

### 2.1. Eligibility Criteria

Studies were considered eligible if they met the following inclusion criteria: original research articles reporting clinical studies in human participants with multiple sclerosis; published in the English language. The literature search covered studies published between January 2020 and September 2025, corresponding to the most recent evidence available at the time the review was conducted. Studies were excluded if they were animal or in vitro studies, review-type publications, or not focused on MS biomarkers. Only studies with retrievable full text were included to enable data extraction and quality assessment; no restriction was applied based on open-access status.

### 2.2. Search Strategy

A comprehensive literature search was conducted across three major electronic databases: Web of Science, PubMed, and Scopus. The primary search terms utilized to identify these studies included combinations of “Multiple Sclerosis” AND (“Neurofilament Light Chain” OR “GFAP” OR “Interleukins”); “Neuroinflammation” AND “Multiple Sclerosis” AND “Biomarkers” “Oxidative Stress Biomarkers” AND “Multiple Sclerosis Progression”.

### 2.3. Document Selection

The initial search yielded a total of 605 articles. Duplicates were identified and removed using the Zotero reference management software (version 7.0.32), followed by a manual verification process to ensure accuracy. This resulted in the removal of 31 duplicate records, leaving 574 unique articles for screening.

These articles were subjected to a rigorous screening process based on title and abstract. After the initial screening and subsequent full-text assessment against the eligibility criteria, 546 articles were excluded. Ultimately, 28 studies were identified as meeting all inclusion criteria. Two independent reviewers screened titles and abstracts against the eligibility criteria. Full texts of potentially eligible articles were then assessed independently by the same reviewers. Disagreements at any stage were resolved through discussion; if consensus was not reached, a third reviewer decided.

### 2.4. Data Collection

Data extracted from each study consisted of author information, year of publication, cohort characteristics, biomarker class, proposed biological pathway, main clinical or MRI outcomes, treatment-related findings, and the risk of bias assessment.

## 3. Results

### 3.1. Study Characteristics

**Figure 1:**
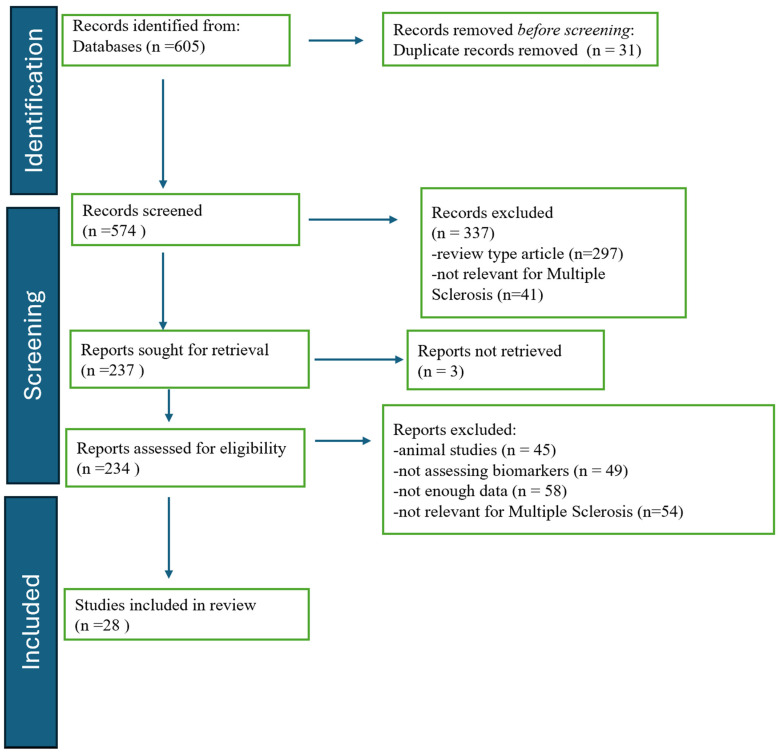
PRISMA 2020 flow diagram of study identification, screening, eligibility, and inclusion.

The database search identified 605 articles. After removing 31 duplicates prior to screening, 574 records remained for title/abstract screening. During this stage, 337 records were excluded, primarily because they were review-type publications (*n* = 297) or not relevant to MS (*n* = 41), as shown in the diagram (Figure 1). The remaining 237 reports were sought for full-text retrieval; 3 reports could not be retrieved. Consequently, 234 full-text articles were assessed for eligibility. Of the full-text articles assessed for eligibility, 206 studies were excluded for the following reasons: animal or in vitro studies (*n* = 45); studies not assessing fluid biomarkers relevant to multiple sclerosis (*n* = 49); insufficient data for extraction (*n* = 58), including studies lacking quantitative biomarker measurements, studies not reporting associations with clinical or imaging outcomes, or studies with incomplete methodological reporting; and studies not directly relevant to multiple sclerosis pathophysiology or clinical outcomes (*n* = 54), such as studies focusing on other neurological diseases or unrelated biological pathways.

The study populations were heterogeneous, represented by all phenotypes of multiple sclerosis: Relapsing-Remitting MS (RRMS), Secondary-Progressive MS (SPMS), Primary Progressive MS (PPMS), and the earliest detectable stages represented by Clinically Isolated Syndrome (CIS) and Radiologically Isolated Syndrome (RIS). The combined cohort included 6365 patients with MS (approximately 80% of total participants) and 1410 controls, which included healthy volunteers, patients with Non-Inflammatory Neurological Diseases (NIOND), and other inflammatory conditions such as NMOSD (Neuromyelitis optica spectrum disorders) and MOGAD (Myelin Oligodendrocyte Glycoprotein Antibody-associated Disease) (Table 1).

The quality of cohort and case–control included studies was assessed using Newcastle–Ottawa Scale (NOS) that evaluates three domains: selection (maximum 4 stars), comparability (maximum 2 stars), and outcome (maximum 3 stars), resulting in a total score of 0–9 stars (17). Studies were categorized as low risk (7–9 stars), moderate risk (6 stars), or high risk (<5 stars). The NOS rating and justifications are summarized in Table 2.

For analytical cross-sectional studies, the JBI Critical Appraisal Checklist for Analytical Cross Sectional Studies (eight items) was used, and studies were graded as low (7–8/8), moderate (4–6/8), or high (0–3/8) based on the number of items judged “Yes,” with particular attention paid to measurement validity, identification/control of confounders, and appropriateness of statistical analysis [49,50]. For randomized trial evidence, including post hoc analyses of RCT datasets, the Cochrane RoB 2 tool was used, producing overall judgments of low risk/some concerns/high risk based on domain-level assessment (randomization process, deviations from intended interventions, missing outcome data, outcome measurement, and selective reporting) [51]. For non-randomized interventional studies (single-arm open-label trials), ROBINS-I was used [52]. Finally, for diagnostic accuracy studies, QUADAS-2 was applied and summarized using its standard qualitative judgments [53]. For non-cohort designs, including post hoc analyses of randomized controlled trials and single-arm interventional studies, ratings of “Some Concerns” were assigned when methodological limitations could introduce potential bias, such as exploratory analyses not pre-specified in the original trial protocol, the possibility of selective outcome reporting, or the absence of a control group, which limits the ability to distinguish treatment effects from natural disease variability. Table 3 outlines the risk of bias for non-cohort studies.

### 3.2. Neurofilament Light Chain (NfL): Marker of Acute Neuroaxonal Injury

NfL was the most frequently investigated biomarker, analyzed in 13 of the 28 studies (46.42%) [22,25,29,34,38,39,40,41,42,43,44,45,47]. Of these, nine articles reported a positive association between elevated NfL levels and acute inflammatory disease activity, relapses, or disability worsening [25,29,38,39,40,41,42,44,45]. These studies consistently reported a strong link between elevated NfL levels and acute inflammatory disease activity and relapse-associated worsening (RAW). Positive correlations with clinical relapses (*p* = 0.044) [39], gadolinium-enhancing lesions (*p* = 0.016) [42], and higher T2 lesion burden (*p* = 0.046) [42] were reported [39,42]. Prognostically, baseline sNfL predicted inflammatory-associated worsening (IAW) with hazard ratios (HR) around 2.1 [29], discriminated aggressive from benign RRMS phenotypes (AUC = 0.77) [25], and forecasted long-term disability (AUC 0.70) [25,29,45]. Higher NfL also correlated with MRI measures of acute white matter injury, including decreased fractional anisotropy (ρ = −0.487, *p* = 0.010) [40,42].

In contrast, the evidence from four studies indicates that NfL is not a reliable marker for progression independent of relapse activity (PIRA) in the absence of ongoing inflammation [22,34,43,47]. In cohorts with non-active progressive MS, sNfL showed no association with confirmed disability progression (adjusted HR 1.11, ρ = 0.488) [43] and only a weak correlation with GFAP, a marker of astrogliosis (r = 0.2–0.3) [34,43]. Furthermore, stool NfL, a novel non-invasive measure, showed no discriminative value between MS phenotypes and correlation with disability change [22].

These findings show that NfL is a good dynamic biomarker of acute neuroaxonal activity but not of smoldering, compartmentalized neurodegenerative progression.

### 3.3. Glial Fibrillary Acidic Protein (GFAP): Marker of Astrogliosis

GFAP was analyzed in 14 of the 28 articles [21,22,25,29,34,36,38,39,40,41,42,43,45,47]. Elevated sGFAP was a specific predictor of non-active PIRA (HR = 3.19, *p* < 0.001) [29]. Increased concentrations were also linked to confirmed disability worsening in patients with progressive forms of MS (HR = 1.71, *p* = 0.004; HR = 2.88, *p* = 0.016) [42,43]. It correlated with neurodegenerative MRI outcomes, including whole brain volume loss (*p* < 0.0001) [36] and retinal layer thinning (*p* < 0.001) [36,41]. Moreover, stool glial fibrillary acidic protein (st-GFAP) was significantly elevated in progressive MS versus RRMS (*p* ≤ 0.0001) and correlated with longitudinal EDSS worsening, highlighting its potential as a non-invasive marker [22]. The link between GFAP and compartmentalized CNS processes was further supported by its strong association with complement activation (*p* < 0.0001) [21].

On the other hand, three studies were not concordant [34,39,47]. In a strictly non-active SPMS cohort, Jiang et al. found no association between serum GFAP and lesion volume change over 96 weeks [34]. Oset et al. reported no meaningful correlations between serum GFAP and cognitive tests (SDMT) [39]. Carmona et al. found no correlation between GFAP and selected cytokines [47].

Taken together, however, the predominant signal across studies supports GFAP as a marker of astrogliosis and silent progression rather than peripheral inflammatory activity. This evidence places GFAP as a key biomarker of astrogliosis, silent progression, and brain atrophy, especially when inflammatory activity is low.

### 3.4. Immunoglobulins, Complement Factors, Cytokines, and Cellular Subsets

Oligoclonal bands (OCBs), free light chains, complement factors, and autoantibodies were analyzed in four articles [21,24,28,29].

Oechtering et al. provided one of the clearest links between innate immune activation and structural damage: doubling of CSF complement components was associated with accelerated annual brain volume loss (C4a −0.24%/year; Ba −0.22%/year; both *p* < 0.0001) [21]. Complement activation also related to inflammatory MRI activity (higher odds of contrast-enhancing lesions for Ba, OR 3.32; *p* = 0.0024) and correlated with astroglial injury as reflected by GFAP (C1q doubling associated with ~40% higher GFAP, *p* < 0.0001) [21].

Monreal et al. reported that LS-OCMB positivity was linked to a more inflammatory/aggressive course and higher NfL levels, supporting the relevance of humoral immunity to inflammatory-associated worsening [29]. Zhang et al. found phenotype- and geography-linked variability in OCB prevalence, and Hegen et al. reported strong correlations between the kappa free light chain (κ-FLC) index and IgG index (r = 0.80, *p* < 0.001), supporting its diagnostic utility in progressive phenotypes [24,28].

Cytokines were investigated in three articles [23,30,37]. Zhu et al. linked MBP-driven IL-17 to relapse activity in women (IL-17 vs. ARR *p* = 0.01) and reported multiple age-related cytokine associations [23]. Shinoda et al. reported strong inverse correlations between baseline CD20dim cytokine+ subsets and treatment-induced changes (IL-17A r = −0.8808, *p* < 0.0001; IFN γ − r = −0.7548, *p* < 0.0001) and showed B-cell cytokine polarization with IL-10+ B cells increased (*p* < 0.01) and IL-6 + B cells decreased (*p* < 0.001) [30]. Sy et al. showed dose-dependent reductions in serum cytokines with 12 g N-acetylglucosamine (GlcNAc) (IL-6, *p* = 0.002; IL-17, *p* = 0.004; IFN-γ, *p* = 0.019) and a transient decrease in Il-10 (*p* = 0.013) during treatment [37].

Cellular subsets were analyzed in three articles [27,30,35] and are presented in Table 4.

### 3.5. Oxidative Stress and Additional Emerging Biomarkers

Oxidative and nitrosative stress markers were analyzed in three articles [31,32,46]. Marček et al. reported a pro-oxidative shift in RRMS versus controls, with higher lipid peroxidation and protein oxidation markers (TBARS and AOPP, both *p* < 0.0001) alongside a reduced antioxidant capacity (TAC *p* < 0.0001; FRAP *p* = 0.0013) [32]. In Räuber et al., nitric oxide metabolites (NOx) in CSF correlated strongly with disability (R^2^ = 0.7494, *p* < 0.0001), supporting compartment-specific associations between metabolic stress and clinical severity [46]. Brichette-Mieg et al. identified redox-linked proteins associated with disability (PRDX6 positively associated with EDSS, *p* = 0.010) [31].

Emerging biomarkers included extracellular vesicle-associated markers Gas6/TAM pathway components and stool-derived biomarkers [22,33,48]. Stool GFAP, evaluated by Schwerdtfeger et al., was significantly elevated in progressive MS (*p* < 0.0001) and correlated with longitudinal EDSS worsening (r = 0.53 over 5 years) [22].

Extracellular vesicle (EV) markers examined by Lim Falk et al. showed inverse correlations with NfL z-scores (*p* < 0.05), suggesting potential regulatory or protective roles [33]. Rosenstein et al. reported that higher soluble Tyro3, and Gas6 predicted greater 60-month white matter and myelin content loss and correlated with GFAP and CSF NfL at follow-up, indicating potential relevance of the TAM receptor pathway in long-term tissue degeneration [48].

### 3.6. Clinical and Radiological Correlations: EDSS and MRI

The correlation between biomarker levels and EDSS scores (or confirmed disability worsening) was explicitly analyzed in 22 studies [21,22,24,25,29,30,31,32,34,36,37,38,39,40,42,43,44,45,46,47,48].

#### 3.6.1. Expanded Disability Status Scale (EDSS)

Most studies reported positive correlations or predictive relationships, with the most consistently implicated markers being neurofilament light chain (NfL) and glial fibrillary acidic protein (GFAP) measured in serum/plasma or CSF; several studies also reported associations for other biomarkers (complement components, ecDNA, NOx, immune cell subsets) and for disability progression endpoints (CDP/CDW, RAW/aPIRA). In contrast, a smaller set of studies reported discordant or null findings, including no EDSS association for CSF oligoclonal band status, selected T-cell subsets, hormonal markers, sTWEAK/sTNF-α, and Gas6-related measures (Table 5).

This table summarizes studies reporting associations between fluid biomarkers and clinical disability, highlighting the consistent positive relationship between markers such as neurofilament light chain (NfL) and glial fibrillary acidic protein (GFAP) and disability progression measured by the Expanded Disability Status Scale (EDSS).

#### 3.6.2. Associations of Biomarkers with MRI Metrics

Brain volume/atrophy outcomes with biomarker associations were reported in 7/28 studies [21,36,38,39,40,44,48]. Oechtering et al. demonstrated that complement activation predicted accelerated brain parenchymal fraction decline (C4a −0.24% per year, *p* < 0.0001) [21]. Harris et al. showed that higher GFAP was associated with lower baseline and 12-month whole-brain volume (*p* < 0.0001) [36]. Jakimovski et al. linked higher NfL to increased choroid plexus volume (β = 0.373, *p* = 0.001) [38]. Rosenstein et al. showed that higher Gas6/Tyro3 predicted greater white matter volume loss over 60 months [48].

Abdelhak et al. reported that higher T2 lesion burden (>8 lesions) was associated with elevated NfL (*p* = 0.046) and GFAP (*p* = 0.002). Monreal et al. showed that greater baseline T2 lesion load predicted inflammatory worsening (HR up to 3.98) [42].

Gadolinium enhancement and MRI inflammatory activity.

Gadolinium enhancement (or closely related MRI activity definitions incorporating gadolinium-enhancing lesions) was analyzed in 9/28 studies with extractable comparisons or associations [21,26,30,32,36,39,42,43,46].

NfL was consistently associated with gadolinium-enhancing lesions (*p* = 0.016) [42,43]. Complement activation was also associated with contrast-enhancing lesions (Ba OR 3.32, *p* = 0.0024) [21]. In contrast, oxidative stress markers such as NOx did not significantly differ by gadolinium-enhancing status [46]. The findings are shown in Table 6.

This table summarizes studies reporting biomarkers that did not show significant associations with clinical disability or disease progression in multiple sclerosis. These findings highlight the heterogeneity of biomarker performance and underscore that not all investigated markers consistently reflect disease severity or progression.

### 3.7. Treatment and Treatment Responses

The impact of therapeutic interventions on biomarker levels or the utility of biomarkers in predicting treatment response was evaluated in 8 of the 28 included articles [26,30,33,36,37,44,45,48].

In an AHSCT-treated RRMS cohort, Erngren et al. reported significant reductions in CSF markers linked to innate immune activation and progressive biology [26]. At 1 year post-AHSCT, Galectin-9 decreased from a median (IQR) of 454 (357–553) to 408 (328–495) pg/mL (*p* = 0.0002), GDF-15 from 49 (38–79) to 45 (35–75) pg/mL (*p* = 0.012), and YKL-40 from 100 (54–164) to 58 (43–92) ng/mL (*p* < 0.0001) [26]. Galectin-9 and YKL-40 declined further between year 1 and year 2 (408 → 376 pg/mL, *p* = 0.0009; and 62 → 56 ng/mL, *p* < 0.0001), after which levels were reported as stable. Importantly, these biomarkers did not differ by MRI activity status in this cohort (Gal-9 *p* = 0.19; GDF-15 *p* = 0.081; YKL-40 *p* = 0.41), suggesting modulation that was not simply driven by acute inflammatory MRI activity [26].

Sy et al. evaluated oral GlcNAc added to background glatiramer acetate and reported dose-related biological changes alongside clinical signals [37]. Serum HexNAc increased by 65% in the 6 g cohort and 112% in the 12 g cohort versus baseline [37]. Clinically, 30% of participants improved, with a mean EDSS decrease of 0.52 points [37].

In a relapsing MS trial dataset, Harris et al. reported that baseline plasma GFAP independently predicted on-treatment relapse count through month 12 (multivariable model β = 0.319, *p* = 0.027), supporting baseline GFAP as a risk-stratification marker for subsequent inflammatory activity under therapy [36].

Other studies suggested more limited or absent biomarker separation by treatment category. Juutinen et al. reported no significant between-group differences in sNfL or sGFAP trajectories over 12 months under menopausal hormone therapy [44]. Rosenstein et al. found no convincing differences in biomarker levels when comparing NEDA-3 versus EDA-3 status or low- versus high-efficacy DMT categories [48]. Falk et al. reported no significant differences in plasma extracellular vesicle quantity or composition markers between treatment groups [33].

## 4. Discussion

Emerging evidence indicates that fluid biomarkers in multiple sclerosis do not represent a single, unified disease process but instead reflect biologically distinct components of MS pathology. Across studies, a consistent pattern is observed whereby markers of acute neuroaxonal injury and focal inflammation diverge from those associated with chronic, compartmentalized neurodegeneration. This dissociation offers a mechanistic framework to explain the well-recognized clinical–radiological paradox in MS and clarifies why disability progression may persist despite effective suppression of overt inflammatory activity.

Neurofilament light chain (NfL) consistently tracks acute inflammatory neuroaxonal injury. Elevated NfL levels correlate with relapses, gadolinium-enhancing lesions, and inflammatory lesion burden [54,55,56,57,58,59], decline under high-efficacy disease-modifying therapies [60,61,62,63], and predict relapse risk and MRI activity [29,36,64]. CSF NfL also demonstrates long-term prognostic value for disability worsening [45]. However, its predictive capacity diminishes in non-active progressive MS [34,43], where progression independent of relapse activity (PIRA) predominates. This limitation is mechanistically coherent as NfL reflects acute axonal transection [65], whereas chronic progression is driven by sustained metabolic stress, mitochondrial dysfunction, iron accumulation, and compartmentalized glial activation [66,67,68,69,70]. These processes may cause slow axonal attrition without generating sufficient acute cytoskeletal fragmentation to markedly elevate NfL.

In contrast, glial fibrillary acidic protein (GFAP) appears to capture this smoldering, innate immune-driven pathology more effectively. Elevated GFAP consistently predicts disability progression, brain atrophy, and retinal neurodegeneration, particularly in patients with low inflammatory activity [41,42,43]. Biologically, GFAP quantifies reactive astrogliosis. Astrocytes, which normally regulate blood–brain barrier integrity and metabolic homeostasis [71,72,73,74,75,76,77], undergo phenotypic transformation in progressive MS, losing protective functions and adopting neurotoxic states that amplify oxidative stress, disrupt mitochondrial integrity, and impair remyelination [66,67,78,79,80]. The strong association between GFAP and complement activation [21,81,82] further implicates astrocyte–microglia crosstalk and innate immune signaling in chronic lesion expansion and ongoing tissue loss.

Taken together, the pathways highlighted across studies support a two-axis model of MS biology. Peripheral adaptive immune activation promotes blood–brain barrier disruption, new inflammatory lesions, and acute axonal injury, which are best captured by NfL, whereas compartmentalized CNS innate immune activation involving astrocytes, microglia, complement signaling, oxidative stress, and mitochondrial dysfunction sustains chronic tissue damage and is better reflected by GFAP and related markers. These axes interact rather than operate independently, because recurrent focal inflammation may prime glial reactivity and glial-mediated injury may perpetuate neuroaxonal loss even after relapse activity is clinically suppressed. This integrative framework helps explain why anti-inflammatory therapies rapidly lower NfL, yet progression can continue as long as astroglial and microglial pathology persist.

These observations further support the distinction between biomarkers of acute inflammatory injury and those associated with chronic glial-mediated neurodegeneration.

Markers of microglial activation, such as CHI3L1 (YKL-40), extend this framework by reflecting CNS-resident innate immune activity that bridges reparative and neurotoxic responses [81,83,84,85,86,87,88]. The reduction in CHI3L1 following autologous hematopoietic stem cell transplantation [26] suggests that aggressive immune resetting can dampen this compartmentalized inflammation. Additional biomarkers, including oxidative and nitrosative stress markers (NOx, ecDNA, PRDX6), correlate with disability severity, aligning with experimental data linking reactive oxygen species and mitochondrial failure to axonal energy collapse [67,89,90,91]. Emerging signals, such as stool GFAP [92,93,94] and extracellular vesicle-associated proteins [95,96,97], raise the possibility that systemic or gut–CNS axis mechanisms may contribute to sustained neuroinflammation. The central role of neuroinflammation in driving tissue injury and limiting recovery in CNS disorders has been extensively documented, including ischemic stroke and systemic autoimmune diseases, where persistent glial activation and dysregulated inflammatory signaling contribute to long-term neurodegeneration [98,99,100].

Not all studies were fully concordant. In particular, some cohorts did not find independent associations between GFAP and lesion change, cognition, or selected cytokines [34,39,47], and NfL-disability relationships were weaker in non-active progressive disease [34,43]. These discrepancies likely reflect differences in phenotype composition, inflammatory activity, assay matrix, follow-up duration, and outcome definitions rather than a complete lack of biological relevance. Compared with prior reviews that mainly focused on individual markers or single pathways [11,16,17,18,19], the present synthesis integrates recent human studies across serum, plasma, CSF, and stool to distinguish biomarkers of acute inflammatory activity from those associated with relapse-independent progression and treatment response.

Together, these findings indicate that NfL predominantly reflects focal inflammatory injury, whereas GFAP and related innate immunity markers capture chronic glial-mediated neurodegeneration. Reliance on NfL alone may therefore underestimate ongoing progression in patients with suppressed inflammatory activity. A multi-biomarker strategy integrating inflammatory and glial markers may improve risk stratification, guide treatment escalation, and accelerate the development of neuroprotective therapies specifically targeting progressive MS.

### Limitations and Future Directions

This review has several limitations. The included studies varied substantially in MS phenotype, disease activity, treatment exposure, sample type, assay platform, and outcome definitions, which precluded formal meta-analysis and made direct comparisons across studies more difficult. In addition, because much of the current evidence comes from observational studies and specialized cohorts, further validation in broader and more diverse patient populations would be valuable to reinforce the clinical relevance of these findings. Future studies should prioritize multicenter prospective designs, standardized biomarker assays, harmonized clinical and MRI outcomes, and longitudinal assessment of biomarker-informed treatment decisions.

## 5. Conclusions

Multiple sclerosis cannot be adequately monitored through an inflammation-centric lens alone. NfL captures acute inflammatory neuroaxonal injury, whereas GFAP and related innate immune markers reflect the compartmentalized, glial-driven neurodegeneration underlying relapse-independent progression. Reliance on a single biomarker risks overlooking silent disability accrual. A multidimensional framework integrating axonal and glial signals is essential to improve risk stratification, redefine trial endpoints, and advance truly mechanism-based therapies for progressive MS.

Beyond acute inflammatory activity, the combined interpretation of axonal and glial biomarkers may offer a more complete picture of disease evolution, particularly in relation to silent progression and ongoing neurodegeneration. From a clinical perspective, this integrated approach could improve patient stratification and therapeutic monitoring, although broader implementation will depend on assay standardization and prospective validation.

Collectively, the evidence synthesized in this review supports a biomarker-dissociation model in multiple sclerosis, in which markers of acute inflammatory neuroaxonal injury diverge from those reflecting chronic and progressive neurodegeneration. Integrating complementary biomarkers that capture inflammatory activity, astroglial activation, immune dysregulation, and neurodegenerative damage may therefore improve the early identification of aggressive or progressive disease phenotypes and help guide more personalized therapeutic strategies.

## Figures and Tables

**Table 1:** Characteristics and summary of included articles.

No.	References	Country	Sample Size (N) and Phenotypes	EDSS and MRI Examined	Markers Assessed	Main Findings
1	[21]	Switzerland	N: 239 Pts CIS, RRMS, SPMS, PPMS	EDSS: yes MRI: Volumetric, T2	CSF: C1q, C3a, C4a, NfL, GFAP	CSF complement activation correlates with MRI structural damage and glial injury
2	[22]	USA/Italy	N: 130 Pts, 31 Ctrl RRMS, ProgMS	EDSS: yes MRI: no	Stool: GFAP, NfL	Stool GFAP elevated in progressive MS
3	[23]	USA	N: 669 Pts RRMS, SPMS	EDSS: no MRI: yes	Peripheral blood mononuclear cells (PBMCs): IL-17, IFN-gamma	IL-17 response declines with age in women
4	[24]	China	N: 483 Pts, 880 Ctrl MS (all types)	EDSS: yes MRI: yes	CSF: OCB	OCB prevalence linked to latitude
5	[25]	Spain	N: 50 Pts, 10 Ctrl Benign vs. Aggressive	EDSS: yes MRI: yes	Serum: NfL, GFAP	Baseline NfL predicts long-term disability
6	[26]	Sweden	N: 45 Pts, 32 Ctrl RRMS (AHSCT)	EDSS: yes MRI: yes	CSF: Gal-9, GDF-15, YKL-40	AHSCT reduces progressive MS markers
7	[27]	Brazil	N: 104 Pts, 58 Ctrl RRMS	EDSS: yes MRI: no	PBMC: CD19+GzmB+	Cytotoxic B cells elevated in RRMS
8	[28]	Multicenter	N: 174 Pts PPMS	EDSS: no MRI: no	CSF: K-FLC	K-FLC index has 93% sensitivity for PPMS
9	[29]	Spain	N: 535 Pts Relapsing MS	EDSS: yes MRI: yes	Serum: NfL, GFAP	High sNfL/LS-OCMB predict inflammatory worsening. High sGFAP predicts Non-Active PIRA
10	[30]	USA	N: 58 Pts RRMS, PPMS	EDSS: yes MRI: yes	PBMC: CD20+T cells	CD20+CD8+ T cells migrate to CNS lesions
11	[31]	Spain	N: 98 Pts, 27 Ctrl Untreated MS	EDSS: yes MRI: yes	Serum: PRDX6, MST1	PRDX6/MST1 correlate with disability
12	[32]	Slovakia	N: 51 Pts, 16 Ctrl Naive RRMS	EDSS: yes MRI: yes	Plasma: ecDNA, DNase	High ecDNA correlates with EDSS
13	[33]	Greece	N: 81 Pts, 15 Ctrl RRMS, NMOSD	EDSS: yes MRI: no	Plasma: EVs, NfL, GFAP	EV markers correlate negatively with NfL
14	[34]	Multicenter	N: 264 Pts SPMS (non-active)	EDSS: yes MRI: yes	Serum: GFAP, NfL	sGFAP does not predict progression in Non-Active SPMS
15	[35]	China	N: 50 Pts, 24 Ctrl RRMS, SPMS	EDSS: yes MRI: yes	PBMC: GzmB+ CD8+	Cytotoxic CD8+ T cells expanded in SPMS
16	[36]	Multicenter	N: 2056 Pts Relapsing MS	EDSS: yes MRI: yes	Plasma: GFAP, NfL	Baseline GFAP predicts on-treatment relapses
17	[37]	USA	N: 34 Pts MS on Glatiramer	EDSS: yes MRI: no	Serum: GlcNAc, NfL	GlcNAc reduces inflammation and NfL
18	[38]	USA	N: 202 Pts RRMS, PMS	EDSS: yes MRI: yes	Serum: NfL, GFAP	GFAP predicts choroid plexus expansion
19	[39]	Poland	N: 50 Pts Early RRMS	EDSS: yes MRI: yes	Serum: NfL, GFAP	Serum NfL correlates with disease activity
20	[40]	Denmark	N: 32 Pts, 32 Ctrl Progressive MS	EDSS: yes MRI: yes	Serum: NfL, GFAP	High sNfL correlates with damage; high sGFAP with preserved cortical structure
21	[41]	Austria	N: 116 Pts Relapsing MS	EDSS: yes MRI: yes	Serum: NfL, GFAP	sGFAP predicts retinal thinning
22	[42]	Germany	N: 243 Pts SPMS, PPMS	EDSS: yes MRI: yes	Serum: NfL, GFAP	sGFAP predicts disability in PPMS
23	[43]	USA	N: 257 Pts Progressive MS	EDSS: yes MRI: yes	Serum: NfL, GFAP	sGFAP predicts progression in Non-Active PMS
24	[44]	Finland	N: 16 Pts, 15 Ctrl Menopausal MS	EDSS: yes MRI: yes	Serum: NfL, GFAP	Low estradiol linked to brain atrophy
25	[45]	Canada	N: 60 Pts MS (Baseline)	EDSS: yes MRI: no	CSF/Serum: NfL/GFAP	CSF NfL and GFAP predicts 15-year disability
26	[46]	Germany	N: 117 Pts RRMS, PPMS	EDSS: yes MRI: yes	Serum/CSF: NOx	Serum NOx higher in MS
27	[47]	France	N: 150 Pts, 186 Ctrl RRMS, PMS	EDSS: yes MRI: no	Serum: TWEAK, NfL, GFAP	No correlation found for NfL/GFAP
28	[48]	Sweden	N: 60 Pts, 25 Ctrl RRMS, PMS	EDSS: yes MRI: yes	CSF: Gas 6, NfL, GFAP	High Gas6/Tyro3 predicts myelin loss

**Table 2:** Risk of bias assessment for cohort studies.

No.	Study	Selection (Max ****)	Comparability (Max **)	Outcome (Max ***)	Total Score	Risk Level	Justification
1	[21]	****	**	**	8/9	Low	Representative cohort; valid MRI/biomarker methods; minor attrition
2	[22]	***	**	***	8/9	Low	Clear definitions; controlled for age/sex; secure ELISA method
3	[25]	***	**	***	8/9	Low	Clear phenotypes adjusted for age/EDSS; long follow-up
4	[26]	****	**	***	9/9	Low	Well-defined AHSCT cohort; adjusted analysis
5	[29]	****	**	***	9/9	Low	Large prospective cohort; extensive adjustment; rigorous definitions
6	[30]	***	**	***	8/9	Low	Independent validation cohort; longitudinal; standardized MRI
7	[38]	****	**	***	9/9	Low	Large longitudinal cohort; adjusted for age/sex/BMI; blinded analysis
8	[39]	***	*	**	6/9	Moderate	Prospective; lack of healthy controls for some metrics; short follow-up
9	[40]	***	**	***	8/9	Low	Recruited from trials; healthy controls included; adjusted for treatment
10	[41]	****	**	***	9/9	Low	Well-defined prospective cohort; multivariate adjustment; blinded analysis
11	[42]	***	*	***	7/9	Low	Large prospective PMS cohort; adjusted analysis; no healthy controls in predictive models
12	[43]	****	**	***	9/9	Low	Long-term natural history cohort; extensive adjustment; blinded analysis
13	[44]	**	*	**	5/9	High	Small sample; limited adjustment; open-label intervention
14	[45]	****	**	***	9/9	Low	Rigorous biobank selection; >15 y follow-up; adjusted for multiple covariates
15	[48]	****	**	***	9/9	Low	Prospective; matched controls; multivariate adjustment; blinded analysis

Stars indicate the number of Newcastle–Ottawa Scale (NOS) criteria fulfilled in each domain for cohort studies. Selection was scored from * to **** (maximum 4 stars), Comparability from * to ** (maximum 2 stars), and Outcome from * to *** (maximum 3 stars). In the Selection domain, one star was awarded for each fulfilled item: representativeness of the cohort, appropriate selection/definition of the comparison group or reference population, secure ascertainment of exposure/biomarker status, and confirmation that the outcome of interest was not present at baseline. In the Comparability domain, one star indicated adjustment for the most important confounder, and two stars indicated additional adjustment for other relevant confounders. In the Outcome domain, one star was awarded for appropriate outcome assessment, one for adequate duration of follow-up, and one for completeness/adequacy of follow-up. Thus, more stars indicate lower risk of bias and better methodological quality within that domain.

**Table 3:** Risk of bias assessment for non-cohort studies.

No.	Article	Study Type	Tool	Score	Qualitative Rating	Key Reasons
1	[23]	Analytical cross-sectional	JBI cross-sectional	5/8	Moderate	Cross-sectional temporality: relapse definition includes radiology report; limited confounding adjustment
2	[33]	Analytical cross-sectional biomarker study	JBI cross-sectional	6/8	Moderate	Cross-sectional; disease activity inferred without MRI endpoints; residual confounding possible
3	[36]	Post hoc analysis of Phase 3 RCTs	RoB 2	NA	Some Concerns	Post hoc exploratory models + nominal *p*-values; missing baseline biomarker subset
4	[37]	Open-label single-arm intervention	ROBINS-I	NA	Some Concerns	No control; post hoc/non-blinded EDSS; short duration; imaging not performed
5	[47]	Retrospective analytical cross-sectional	JBI cross-sectional	5/8	Moderate	Cross-sectional; limited confounding control; no longitudinal outcomes; no MRI integration
6	[34]	Post hoc analysis of Phase 3 RCT (ASCEND subset)	RoB 2	NA	Some Concerns	Post hoc restriction to MRI-inactive subset; exploratory analyses; atrophy not assessed
7	[24]	Multicenter cross-sectional	JBI cross-sectional	5/8	Moderate	Cross-sectional; MRI limited to lesion location; potential inter-center heterogeneity
8	[28]	Multicenter diagnostic accuracy	QUADAS-2	NA	Some Concerns	Retrospective pooling + center heterogeneity; strong lab testing; no MRI correlates
9	[35]	Single-center cross-sectional (immune profiling)	JBI cross-sectional	5/8	Moderate	Cross-sectional; small SPMS subgroup; no quantitative MRI outcomes
10	[27]	Single-center cross-sectional (immunophenotyping)	JBI cross-sectional	5/8	Moderate	Cross-sectional; no longitudinal endpoints; no MRI integration
11	[31]	Multicenter cross-sectional (discovery + validation)	JBI cross-sectional	5/8	Moderate	Strong proteomics + validation; untreated cohort; MRI only descriptive (Gd counts), not analyzed
12	[32]	Single-center cross-sectional (RRMS; CSF subset)	JBI cross-sectional	5/8	Moderate	CSF only in subset might cause selection risk; threshold-based lesion subgroup; volumetry described but not analyzed
13	[46]	Single-center cross-sectional (diagnostic biomarker)	JBI cross-sectional	5/8	Moderate	Cross-sectional; MRI activity binary only; very small PPMS CSF subgroup

**Table 4:** Cellular subset correlates MRI activity, disability measures, and disease stage in multiple sclerosis.

No.	Article	Cellular Subset (Measure)	Outcome Compared/Correlated	Effect/Statistic
1	[30]	CD20dim T cells (% Peripheral blood mononuclear cells (PBMCs)) CD20dim CD8+ T cells (% PBMCs) CD20dim CD4+ T cells (% PBMCs)	Baseline gadolinium-enhancing T1 lesions	r = −0.6663, *p* = 0.0004 r = −0.6332, *p* = 0.0009 r = −0.3366, *p* = 0.1077
2	[35]	GzmB+CD8+ T-cell proportion GzmB+CD8+TEMRA (classification performance) GzmB+CD8+T (classification performance) GzmB+CD8+TEM (classification performance) Cut-offs (percent positive)	T25W MSWS-12 9-HPT SPMS vs. RRMS (ROC) SPMS vs. RRMS (ROC cut-offs)	r = 0.651, *p* < 0.001 r = 0.497, *p* = 0.002 r = 0.553, *p* = 0.009 AUC 95.3%, *p* < 0.001 (TEMRA) AUC 94.3%, *p* < 0.001 (CD8+T) AUC 76.6%, *p* = 0.003 (TEM) 35.2% (GzmB+CD8+T); 36.2% (GzmB+CD8+TEM); 53.4% (GzmB+CD8+TEMRA)
3	[27]	CD8+GzmB+ T cells (group comparison) CD19+GzmB+ B cells (group comparison) GzmB concentration in stimulated CD19+ B-cell supernatant	RRMS vs. healthy donors	34.5 vs. 20.8 (mean; 95% CI), *p* < 0.0003 13.6 vs. 1.8 (mean; 95% CI), *p* < 0.0001 368.9 vs. 15.1 (mean; SEM), *p* = 0.0145

**Table 5:** Correlations between biomarker levels and EDSS scores.

	No.	References	Biomarker(s) Analyzed	Finding Regarding EDSS/Disability
Positive correlations	1	[21]	CSF C3a, C4a, NfL	Levels correlated with disease severity
	2	[22]	Stool GFAP	Correlated with baseline EDSS and worsening at 2 years
	3	[25]	Serum NfL	Levels were significantly higher in “aggressive” MS (EDSS > 6) compared to “benign” MS
	4	[29]	Serum NfL, LS-OCMB	Predicted relapse-associated worsening (RAW) and active progression independent of relapse activity (aPIRA)
	5	[31]	Serum PRDX6, MST1, APEH	Positively correlated with EDSS scores
	6	[32]	Plasma extracellular DNA (ecDNA)	Positively correlated with EDSS (r = 0.46)
	7	[35]	Gzm B+CD8+TEMRA cells	Frequency strongly correlated with EDSS (r = 0.627)
	8	[36]	Plasma GFAP (baseline)	Positively associated with month 12 EDSS score
	9	[37]	GlcNAc (supplementation)	Supplementation improved EDSS scores (inverse relationship with severity)
	10	[38]	Serum NfL, GFAP	Levels significantly higher in progressive MS patients (who had higher EDSS) compared to Relapsing-Remitting patients
	11	[39]	Serum NfL (baseline)	Predicted higher EDSS progression
	12	[40]	Serum NfL	Positively correlated with EDSS scores at follow-up (rho = 0.424)
	13	[42]	Serum GFAP(z-scores)	z-score > 3 predicted disability progression in PPMS
	14	[43]	Serum GFAP	Predicted 6-month confirmed disability progression (CDP)
	15	[45]	CSF NfL, CSF GFAP (baseline)	Independently predicted long-term confirmed disability worsening (CDW)
	16	[46]	CSF NOx	Positively correlated with EDSS (R^2^ = 0.7494)
Discordant or null findings	1	[47]	sTWEAK, sTNF-α	No correlation found with EDSS scores
	2	[48]	Gas6, receptors	Not associated with EDSS at baseline or follow-up
	3	[24]	CSF-OCB	No significant difference in EDSS between OCB-positive and OCB-negative patients
	4	[30]	T-cell subsets	No correlation found (EDSS remained stable during the study)
	5	[34]	Serum GFAP (changes)	Changes in levels did not correlate with EDSS changes in non-active SPMS
	6	[44]	Estradiol, FSH	Hormone levels did not correlate with EDSS

**Table 6:** Associations of biomarkers with MRI metrics.

No.	Article	Brain Volume/Atrophy	Lesion Load	Gadolinium Enhancement
1	[21]	BPF decline (annual): per doubling (CSF) C4a −0.24%/y (95% CI −0.31 to −0.16), *p* < 1 × 10^−4^. Ba −0.22%/y (−0.29 to −0.15), *p* < 1 × 10^−4^. C3a −0.13%/y (−0.21 to −0.06), *p* = 0.00024. Bb −0.12%/y (−0.17 to −0.07), *p* < 1 × 10^−4^. C5a −0.07%/y (−0.11 to −0.04), *p* < 1 × 10^−4^. s-C5b9 −0.06%/y (−0.09 to −0.03), *p* < 1 × 10^−4^.	Longitudinal T2 lesion volume: per doubling (CSF): C3a ME 2.19 (1.58–3.04), *p* < 1 × 10^−4^; Ba ME 1.97 (1.26–3.08), *p* = 0.00376; C4a ME 1.79 (1.23–2.60), *p* = 0.00292; s-C5b9 ME 1.20 (1.04–1.38), *p* = 0.01415.	CEL presence: Ba OR 3.32 (1.53–7.21), *p* = 0.00240; C3a OR 2.54 (1.40–4.61), *p* = 0.00224; C5a OR 1.40 (1.03–1.91), *p* = 0.03323; C4a ns OR 1.81 (0.97–3.36), *p* = 0.0623.
2	[25]	Not assessed (no atrophy metrics; “absence of follow-up radiological data” noted as limitation).	No quantitative lesion load: only baseline “radiological activity” and limited ability to assess new T2 lesions (8/48 had prior MRI comparison.	Baseline MRI activity: 5/48 total; 2 bRRMS vs. 3 aRRMS, *p* = 0.349 (Fisher).
3	[29]	Not assessed (no volumetry/atrophy)	Baseline T2 lesion load (categorical) predicts inflammatory worsening (Cox): RAW: 10–50 lesions HR 2.30 (1.08–4.88), *p* = 0.03; >50 lesions HR 3.98 (1.47–10.7), *p* = 0.006. aPIRA: 10–50 HR 3.48 (1.04–11.7), *p* = 0.04; >50 HR 4.98 (1.23–20.2), *p* = 0.02. No association with naPIRA.	MRI activity (new T2 and/or gadolinium-enhancing lesions within 1 year of PIRA event) used to define aPIRA vs. naPIRA. Baseline: ≥1 gadolinium-enhancing lesions in 56.6%, median 1 (0–45).
4	[30]	Not assessed.	Not primary; descriptive subgroup: higher baseline T2 lesion number *p* = 0.011 and T2 lesion volume *p* = 0.050 in those with later MRI activity.	Baseline gadolinium-enhancing lesions (*n* = 24): 10/24 (42%) had ≥1.
5	[36]	WBV: baseline GFAP vs. baseline WBV β = −0.0012 (SE 0.0001), *p* < 0.0001. Baseline GFAP vs. WBV at Month 12 β = −3.6935 cm^3^ (SE 0.4924), *p* < 0.0001.	Baseline GFAP vs. baseline T2 lesions β = 0.1776 (SE 0.0127), *p* < 0.0001. Baseline GFAP vs. new/enlarging T2 lesions over 12 months β = 0.5688 (SE 0.0823), *p* < 0.0001.	Baseline GFAP vs. baseline GdE lesions β = 0.1561 (SE 0.0125), *p* < 0.0001. Baseline GFAP vs. GdE lesions at Month 12 β = 0.9835 (SE 0.1448), *p* < 0.0001.
6	[40]	DTI microstructure: NfL ↔ NAWM: FA ρ = −0.487 *p* = 0.010; MD ρ = 0.547 *p* = 0.003; GLM log2(NfL) → FA β = −0.006 *p* = 0.013; log2(NfL) → MD *p* = 0.004; GFAP ↔ CGM: FA ρ = 0.592 *p* = 0.001; MD ρ = −0.396 *p* = 0.041; GLM log2(GFAP) → FA *p* = 0.009; →MD *p* = 0.015. PBVC median −1.50% (−0.50%/y).	Lesion volume increased (median +48.38 μL/y), *p* = 0.024; PASAT worsening correlated with increasing T2 lesion volume (ρ = −0.508 *p* = 0.0049 cross-sectional; ρ = −0.408 *p* = 0.031 longitudinal). No significant NfL/GFAP association with new/enlarging lesion counts.	Not assessed.
7	[39]	Linear atrophy measures: BCR increased 0.125 → 0.138 (Z = 4.66, *p* < 0.001); TVW 3.95 → 4.00 mm (Z = 2.84, *p* = 0.005). SDMT correlates: BCR R = −0.32 *p* = 0.025; TVW R = −0.28 *p* = 0.049. TVW predicts impaired processing speed β = 0.720 *p* = 0.030; AUC = 0.764 *p* = 0.004; cutoff 5.2 mm.	T2 lesion number: no NEDA vs. EDA difference *p* = 0.138.	Baseline gadolinium-enhancing lesions: no NEDA vs. EDA difference, *p* = 0.277.
8	[38]	Choroid plexus (CP) volume: follow-up CP volume assoc. NfL β = 0.373 *p* = 0.001; OPN β = −0.230 *p* = 0.020 (R^2^ 9.5% → 19%, *p* < 0.001). Baseline predictors of 5-y CP % expansion: GFAP β = 0.277 *p* = 0.004; FLRT2 β = −0.226 *p* = 0.014; in pwPMS FLRT2 β = −0.462 *p* = 0.010.	Not assessed.	Not assessed.
9	[43]	Not assessed.	MRI activity (new/enlarging T2 lesions) associated with higher sNfL: adjusted β = 1.17 (1.01–1.36), *p* = 0.042. sGFAP ns β = 1.05 (0.93–1.18), *p* = 0.457.	Gadolinium-enhancing lesions within 30 days of baseline: higher sNfL adjusted β = 1.46 (1.08–1.96), *p* = 0.014. sGFAP ns β = 0.89 (0.70–1.14), *p* = 0.357.
10	[42]	Not assessed (MRI dataset explicitly limited; no atrophy metrics).	T2 lesion count category (>8 lesions) associated with higher GFAP Z (*p* = 0.002) and higher NfL Z (*p* = 0.046).	Recent CEL presence associated with higher NfL Z (*p* = 0.016) but not GFAP Z (*p* = 0.961).
11	[44]	Estradiol vs. WBV: r = 0.76 *p* = 0.003. Low estradiol independently associated with lower WBV: β = 340.8 mL (102.4–579.3), *p* = 0.01; remained significant adjusting for disease duration *p* = 0.009. WBV change +1.9% *p* = 0.084.	Estradiol vs. WM lesion volume: r = −0.69 *p* = 0.008; not independent after age adjustment *p* = 0.12. Lesion volume changes +1.0 mL *p* = 0.16.	Assessed descriptively: no gadolinium-enhancing lesions at 12 months; no association analyses.
12	[48]	WM volume loss over 60 months: baseline Tyro3 β = 25.5 mL (6.11–44.96), *p* = 0.012; Gas6 β = 11.4 mL (0.42–22.4), *p* = 0.042. No association with GM or BPF change.	Quantitative myelin content (MyC) change: Tyro3 β = 7.95 mL (1.84–14.07), *p* = 0.012; Gas6 β = 4.4 mL (1.04–7.75), *p* = 0.012. remyelination subgroup had lower baseline Tyro3 *p* = 0.033 and Gas6 *p* = 0.014.	Not associated: biomarkers did not associate with contrast-enhancing lesions.
13	[34]	Not assessed.	Baseline sGFAP associated with baseline T2 lesion volume: pooled *p* < 0.001; natalizumab *p* < 0.001. placebo (sNfL) *p* = 0.002. No association with T2/T1 lesion volume change or SEL volume/change (all *p* > 0.05).	Not assessed by design: inclusion required no baseline/follow-up Gd+ lesions.
14	[24]	Not assessed.	Lesion location association only: periventricular lesions more frequent in OCB+ vs. OCB− (93.6% vs. 86.5%, *p* = 0.017).	Not assessed.
15	[31]	Not assessed.	Not assessed.	Descriptive only: Gd-enhancing T1 lesions RRMS 9/38; SPMS 1/21; PPMS 5/21.
16	[32]	Protocol describes atrophy/volumetry availability (icobrainMS + visual scales) but no reported associations linking ecDNA/mtDNA/DNase to atrophy metrics.	High lesion load > 9 T2 lesions: CSF mtDNA 4326.11 GE/mL (IQR 32,643.85) vs. 1103.7 (IQR 1326.54), *p* = 0.043. Descriptive: T2 lesion load 24.043 ± 16.748; FLAIR lesion volume 4.650 ± 4.680 mL; T1 lesion volume 2.892 ± 3.349 mL.	Gadolinium-enhancing lesions: CSF mtDNA 38,260.01 GE/mL (IQR 112,818.96) vs. 1520.15 (IQR 3370.11), *p* = 0.041. CSF ecDNA 45.66 ng/mL (IQR 50.08) vs. 8.14 (IQR 5.16), *p* = 0.031.
17	[46]	Not assessed.	Not assessed.	Tested but ns: no differences in serum/CSF NOx between RRMS with vs. without contrast-enhancing lesions.
18	[26]	Not assessed.	Not assessed (MRI event defined but no lesion loads quantified). MRI activity defined as new T2 lesions >3 mm, but lesion load not quantified.	Gadolinium-enhancing lesions used to define active disease/NEDA; no biomarker differences by activity: Gal-9 *p* = 0.19; GDF-15 *p* = 0.081; YKL-40 *p* = 0.41.

## Data Availability

The original contributions presented in this study are included in the article. Further inquiries can be directed to the corresponding author.

## Supplementary Materials

The following supporting information can be downloaded at: https://www.mdpi.com/article/10.3390/cells15070610/s1, Table S1: PRISMA checklist 2020.

## Abbreviations

AHSCT	Autologous Hematopoietic Stem Cell Transplantation
AOPP	Advanced Oxidation Protein Products
ARR	Annualized Relapse Rate
AUC	Area Under the Curve
Ba	Complement Factor Ba
Bb	Complement Factor Bb
BCR	Brain Central Ratio
BPF	Brain Parenchymal Fraction
BMI	Body Mass Index
CDP	Confirmed Disability Progression
CDW	Confirmed Disability Worsening
CEL	Contrast-Enhancing Lesions
CGM	Cortical Gray Matter
CHI3L1	Chitinase 3-Like 1
CIS	Clinically Isolated Syndrome
CNS	Central Nervous System
CSF	Cerebrospinal Fluid
DTI	Diffusion Tensor Imaging
DMT	Disease-Modifying Therapy
DNase	Deoxyribonuclease
ecDNA	Extracellular DNA
EDSS	Expanded Disability Status Scale
EDA	Evidence of Disease Activity
ELISA	Enzyme-Linked Immunosorbent Assay
EV	Extracellular Vesicle
FA	Fractional Anisotropy
FLAIR	Fluid-Attenuated Inversion Recovery
FRAP	Ferric-Reducing Ability of Plasma
FSH	Follicle-Stimulating Hormone
Gal-9	Galectin-9
Gas6	Growth Arrest-Specific 6
GDF-15	Growth Differentiation Factor 15
GFAP	Glial Fibrillary Acidic Protein
GzmB	Granzyme B
HR	Hazard Ratio
IAW	Inflammatory-Associated Worsening
IgG	Immunoglobulin G
IL	Interleukin
IQR	Interquartile Range
K-FLC	Kappa Free Light Chains
LS-OCMB	Lipid-Specific IgM Oligoclonal Band
MBP	Myelin Basic Protein
MD	Mean Diffusivity
MRI	Magnetic Resonance Imaging
MS	Multiple Sclerosis
MST1	Macrophage-Stimulating 1
mtDNA	Mitochondrial DNA
NAWM	Normal-Appearing White Matter
NEDA	No Evidence of Disease Activity
NfL	Neurofilament Light Chain
NMOSD	Neuromyelitis Optica Spectrum Disorder
NOx	Nitric Oxide Metabolites
NOS	Newcastle–Ottawa Scale
OCB	Oligoclonal Band
OPN	Osteopontin
PBMC	Peripheral Blood Mononuclear Cells
PBVC	Percentage Brain Volume Change
PDDS	Patient-Determined Disease Steps
PIRA	Progression Independent of Relapse Activity
PPMS	Primary Progressive Multiple Sclerosis
PRDX6	Peroxiredoxin 6
PRISMA	Preferred Reporting Items for Systematic Reviews and Meta-Analyses
RAW	Relapse-Associated Worsening
RIS	Radiologically Isolated Syndrome
RoB	Risk of Bias
ROBINS-I	Risk Of Bias In Non-Randomized Studies of Interventions
ROC	Receiver Operating Characteristic
RRMS	Relapsing-Remitting Multiple Sclerosis
SDMT	Symbol Digit Modalities Test
SEL	Slowly Expanding Lesions
SEM	Standard Error of the Mean
SPMS	Secondary Progressive Multiple Sclerosis
sNfL	Serum Neurofilament Light Chain
sGFAP	Serum Glial Fibrillary Acidic Protein
TBARS	Thiobarbituric Acid Reactive Substances
T25FW	Timed 25-Foot Walk
T2	T2-Weighted MRI Sequence
TAC	Total Antioxidant Capacity
TEM	Effector Memory T Cells
TEMRA	Terminally Differentiated Effector Memory T-Cells Re-expressing CD45RA
TVW	Third Ventricle Width
WBV	Whole-Brain Volume
YKL-40	Chitinase 3-Like Protein
